# The first description of the nymphal stages of *Hoplopleura longula* (Psocodea: Anoplura: Hoplopleuridae) from the harvest mouse *Micromys minutus* (Rodentia: Muridae)

**DOI:** 10.3897/BDJ.9.e63747

**Published:** 2021-04-19

**Authors:** Paulina Kozina, Joanna N. Izdebska, Rafal Kowalczyk

**Affiliations:** 1 University of Gdansk, Faculty of Biology, Gdansk, Poland University of Gdansk, Faculty of Biology Gdansk Poland; 2 Mammal Research Institute Polish Academy of Sciences, Bialowieza, Poland Mammal Research Institute Polish Academy of Sciences Bialowieza Poland

**Keywords:** mammals, parasite, rodents, sucking lice, taxonomy

## Abstract

**Background:**

Despite the widespread belief that an extensive body of knowledge exists for the sucking lice (Anoplura), some of their common, Eurasian or even cosmopolitan species still lack complete taxonomic descriptions, especially those for their nymphal stages. This applies especially to the most common rodent parasites: the lice of the genus *Hoplopleura*. In Europe, only two of the five most common *Hoplopleura* species have full taxonomic characteristics with a description of the nymphal stages. This study enriches the current state of knowledge for another species, *Hoplopleura longula* and presents the first description of its nymphal stages.

The study includes five rare louse specimens (two nymphs I, one nymph II, two nymphs III) of *H. longula* collected from 63 Eurasian harvest mice *Micromys minutus*. The collected lice were fixed and preserved in 70% ethyl alcohol solution and then placed in polyvinyl-lactophenol to form total preparations.

**New information:**

Only two of the five species found in Eurasia (*H. acanthopus*, *H. affinis*, *H. captiosa*, *H. edentula* and *H. longula*) have been given full taxonomic descriptions, including immature stages. This paper presents a description of the nymphal stages of *H. longula* (described for the first time).

## Introduction

Sucking lice (Psocodea: Anoplura) are obligatory, permanent ectoparasites of mammals. Lice complete their entire lifecycle on their host and it includes: egg, three nymphal stages (nymph I, nymph II, nymph III) and an adult stage (imago). Some past research on this group has focused on their relationship with humans (e.g. Buxton 1941, Müller 1949, Piotrowski 1961, Fisher and Morton 1970, Downs et al. 1999, Roux and Raoult 1999, Levot 2000, Robinson et al. 2003, Izdebska 2014, Izdebska et al. 2018) or livestock (Piotrowski 1998, Mey 2003) and their potential for disease transmission (e.g. Piotrowski and Wegner 1976, Fagir et al. 2014). In comparision, there have been relatively few studies examining the sucking lice of wild fauna. As such, only fragmentary knowledge exists about certain species.

Approximately 70% of Anoplura are associated with rodents (Rodentia), the most abundant group of mammals in terms of species (Durden and Musser 1994, Baker et al. 2002, Kim 2006). The family Hoplopleuridae (Psocodea: Anoplura) is the most speciose group parasitising the Rodentia. Amongst the family, most representatives are in the genus *Hoplopleura*, which contains 154 species distributed around the world. The following five are most widespread in Europe - four of them being common in Eurasia: *H. acanthopus* (Burmeister, 1839), *H. affinis* (Burmeister, 1839), *H. edentula* and *H. longula* Neumann, 1909 and one is probably cosmopolitan: *H. captiosa* Johnson, 1960 (Durden and Musser 1994).

Numerous publications concerning the ectoparasites of European fauna mention the presence of lice on rodents and insectivores (e.g. Krištofik 1999, Izdebska and Fryderyk 2010, Krcmar and Trilar 2017); however, concise taxonomic work concerning the genus *Hoplopleura* are scarce (Kim 1966, Johnson 1972, Weaver and Barton 2008) and the nymphal stages have been rarely described (Cook and Beer 1959, Johnson 1960, Kim 1966, Wegner 1966a). Of the species found in Eurasia, only two have been given full taxonomic descriptions, including their immature stages. This paper presents the first characterisation of the nymphal stages of *Hoplopleura longula*.

## Materials and methods

Five louse specimens were included in this study (two nymphs I, one nymph II and two nymphs III). These were isolated from four (out of 63 examined individuals) Eurasian harvest mice *Micromys minutus* (Pallas, 1771), obtained from the Scientific Collection of the Mammal Research Institute Polish Academy of Sciences in Bialowieza (Table 1). The hosts originated from the area of the Bialowieza National Park and the material was collected in the period 1949-1972. The specimens found were deposited in the Collection of Extant Invertebrates, University of Gdansk, Department of Invertebrate Zoology and Parasitology, Gdansk, Poland, UGDIZP (Table 1) (Zhang 2018).

Lice were collected from dead rodent specimens by combing the fur with tweezers and the specimens were fixed and preserved in 70% ethyl alcohol. The specimens, intended for analysis of morphological traits, were immersed in polyvinyl-lactophenol to develop total preparations for light microscope examination (Kadulski and Izdebska 2006).

Topographic preferences were observed. The entire body surface area of the test hosts was analysed and all locations were marked; this allowed for a complete (intensive) analysis of the topography.

**Figure 1.**
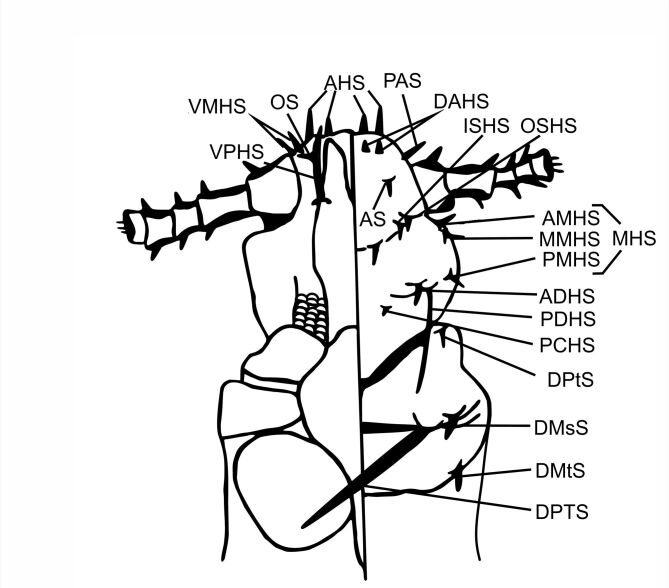
*Hoplopleura* ventral (left) and dorsal (right) view of head and thorax.

The names and abbreviations of individual setae or body parts are provided following Kim (1966) and Kim and Ludwig (1978) (Fig. 1).

Abbreviations of head and thorax:

- **ADHS** accessory dorsal head setae;
- **AHS** apical head setae;
- **AS** antennal setae;
- **CS** clypeal setae;
- **DAHS** dorsal anterior head setae;
- **DMsS** dorsal mesothoracic setae;
- **DMtS** dorsal metathoracic setae;
- **DPTS** dorsal principal thoracic setae;
- **DPtS** dorsal prothoracic setae;
- **ISHS** inner sutural head setae;
- **MHS** marginal head setae;
- **AMHS** anterior marginal head setae;
- **MMHS** middle marginal head setae;
- **PMHS** posterior marginal head setae;
- **OS** oral setae;
- **OSHS** outer sutural head setae;
- **PAS** preantennal setae;
- **PCHS** posterior central head setae;
- **PDHS** posterior dorsal head setae;
- **VMHS** ventral marginal head setae;
- **VPHS** ventral principal head setae.

Abbreviations of abdomen:

- **AnS** anal setae;
- **MAS** major abdominal setae.

## Taxon treatments

### Hoplopleura longula

Neumann, 1909

D8E3045B-4432-5473-9A0D-8B006A12B951

**Type host**: Micromys minutus (Pallas, 1771)
**Other hosts**: Micromys glareolus Schreber, 1780 in Krištofik and Lysy (1992);
Microtus arvalis Pallas, 1778 and *Sorex araneus* Linnaeus, 1758 in Wegner (1966b)

#### Materials

- **Type status:** Other material. **Occurrence:** lifeStage: 1 nymph first instar; **Taxon:** scientificName: *Hoplopleura longula* Neumann, 1909; kingdom: Animalia; phylum: Arthropoda; class: Insecta; order: Psocodea; family: Hoplopleuridae; genus: Hoplopleura; **Location:** continent: Europe; country: Poland; locality: area of the Bialowieza National Park; verbatimCoordinates: 52°45'23.3"N 23°52'23.6"E; georeferenceProtocol: GPS; **Identification:** identifiedBy: Kozina P.; **Event:** samplingProtocol: host *Micromys minutus*; eventDate: 20-09-1949; **Record Level:** institutionCode: UGDIZPMMmHHl1N1
- **Type status:** Other material. **Occurrence:** lifeStage: 1 nymph first instar and 1 nymph second instar; **Taxon:** scientificName: *Hoplopleura longula* Neumann, 1909; kingdom: Animalia; phylum: Arthropoda; class: Insecta; order: Psocodea; family: Hoplopleuridae; genus: Hoplopleura; **Location:** continent: Europe; country: Poland; locality: area of the Bialowieza National Park; verbatimCoordinates: 52°45'23.3"N 23°52'23.6"E; georeferenceProtocol: GPS; **Identification:** identifiedBy: Kozina P.; **Event:** samplingProtocol: host *Micromys minutus*; eventDate: 08-12-1949; **Record Level:** institutionCode: UGDIZPMMmHHl2N1, UGDIZPMMmHHl1N2
- **Type status:** Other material. **Occurrence:** lifeStage: 2 nymph third instar; **Taxon:** scientificName: *Hoplopleura longula* Neumann, 1909; kingdom: Animalia; phylum: Arthropoda; class: Insecta; order: Psocodea; family: Hoplopleuridae; genus: Hoplopleura; **Location:** continent: Europe; country: Poland; locality: area of the Bialowieza National Park; verbatimCoordinates: 52°45'23.3"N 23°52'23.6"E; georeferenceProtocol: GPS; **Identification:** identifiedBy: Kozina P.; **Event:** samplingProtocol: host *Micromys minutus*; eventDate: 07-10-1949; **Record Level:** institutionCode: UGDIZPMMmHHl1N3, UGDIZPMMmHHl2N3

#### Description

**Figure 2.**
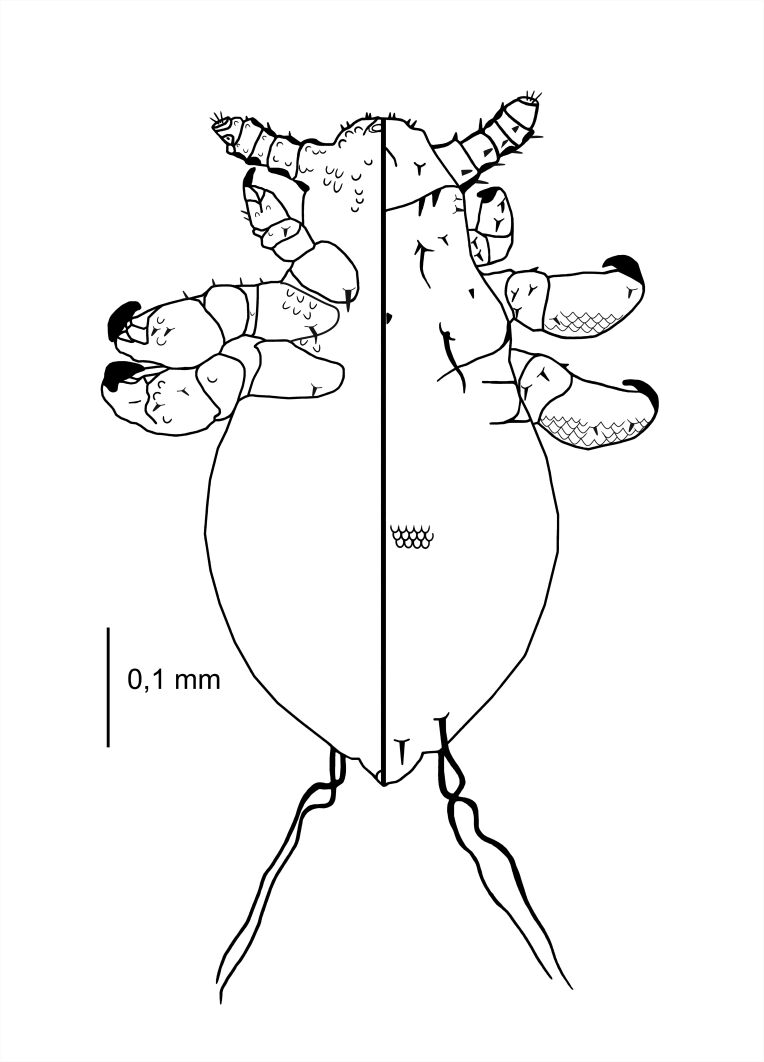
*Hoplopleura longula* nymph I, ventral (left) and dorsal (right) view.

**Figure 3.**
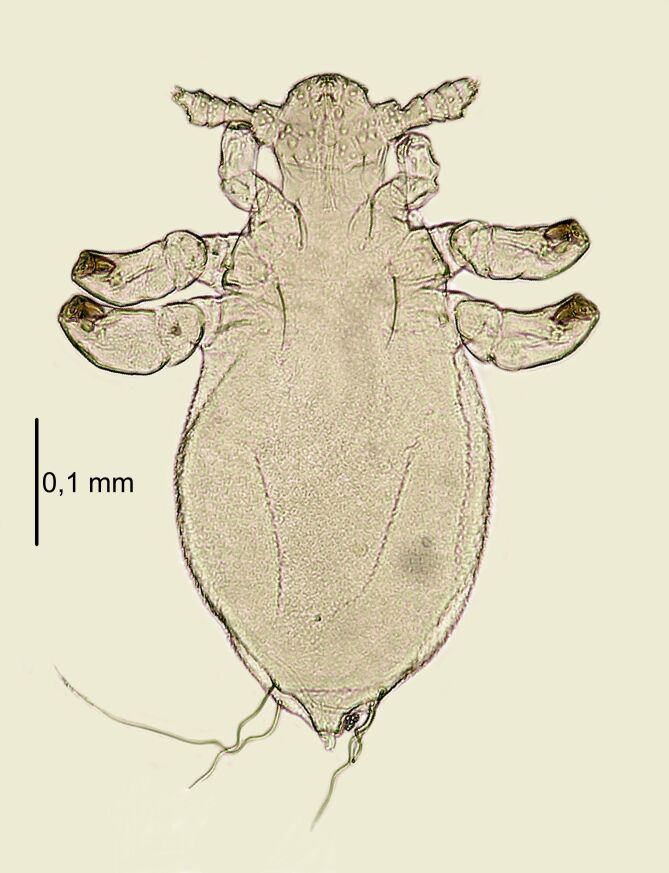
*Hoplopleura longula* nymph I, dorsal view.

**Nymph I** (Figs 2, 3) . Legs large in proportion to the rest of the body, rapid growth with moulting to stage II; body length 0.53 mm (Table 2). **Head**. A poorly-marked line dividing the head and thorax. Ventral side: anterior part with the mouth rather depressed, not pronounced; convex scales around the mouth and in the site of the future gular plate; VMHS, OS and AHS present; VPHS very long, close to 75% of head length. Dorsal side: margins of the head shield poorly outlined; AHS, DAHS, PAS present; OSHS and ISHS present, very distant; ADHS and PDHS present, reaching half of the first thorax segment; a considerable number of convex, U-shaped scales. **Thorax**. Dorsal side: DPTS reaching anterior part of the thorax; DPtS, DMsS and DMtS shifted towards mid-portion of the body. **Abdomen.** Ovoid (directly after hatching: more elongated). MAS four in number; AnS present, two in number.

**Figure 4.**
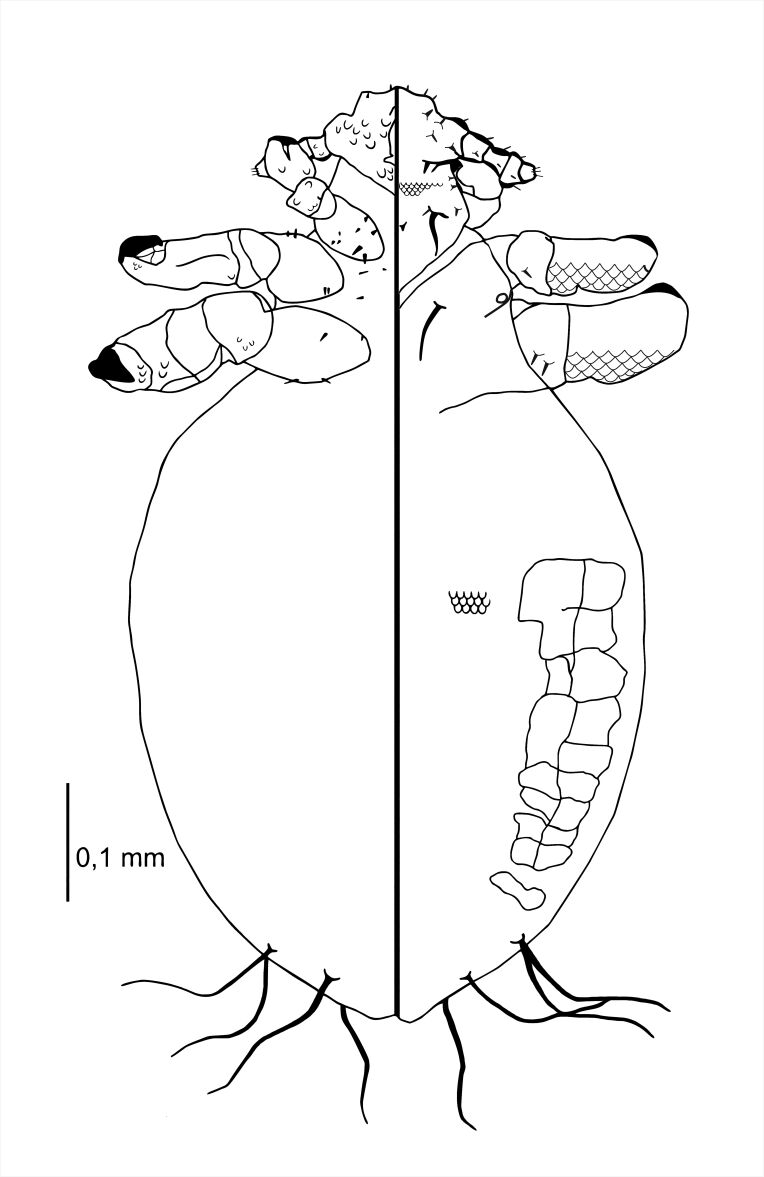
*Hoplopleura longula* nymph II, ventral (left) and dorsal (right) view.

**Figure 5.**
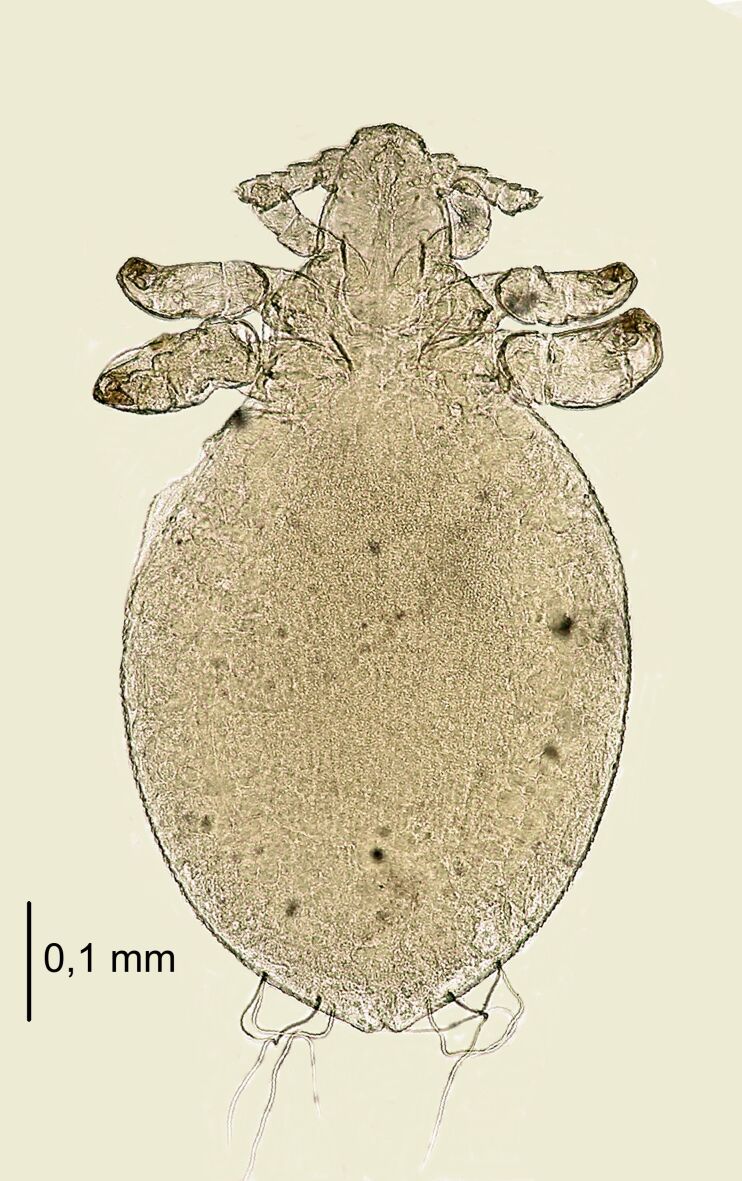
*Hoplopleura longula* nymph II, dorsal view.

**Nymph II** (Figs 4, 5) . Body length: 0.78 mm (Table 2). **Head**. Wider than long; anterior part of the head shield square with smooth margins. Head well pronounced against the thorax (indentation visible at the contact point of both parts). Ventral side: large, concave scales around the mouth; head margins covered imbricately with scales (U-shaped); VMHS, OS and AHS present; VPHS constituting 50% of the head length. Dorsal side: AHS, DAHS and PAS present; ISHS and OSHS closely arranged; PCHS visible; ADHS and PDHS (reaching second segment of the abdomen) present; MHS minute. **Thorax**. Dorsal side: DPTS reaching beginning of the abdomen; pronounced border between the thorax, head and abdomen. **Abdomen**. Disproportionately large relative to the rest of the body, barrel-shaped, densely covered with U-shaped scales; traces of segmentation visible; MAS eight in number. After x-ray, structure of nymph III visible.

**Figure 6.**
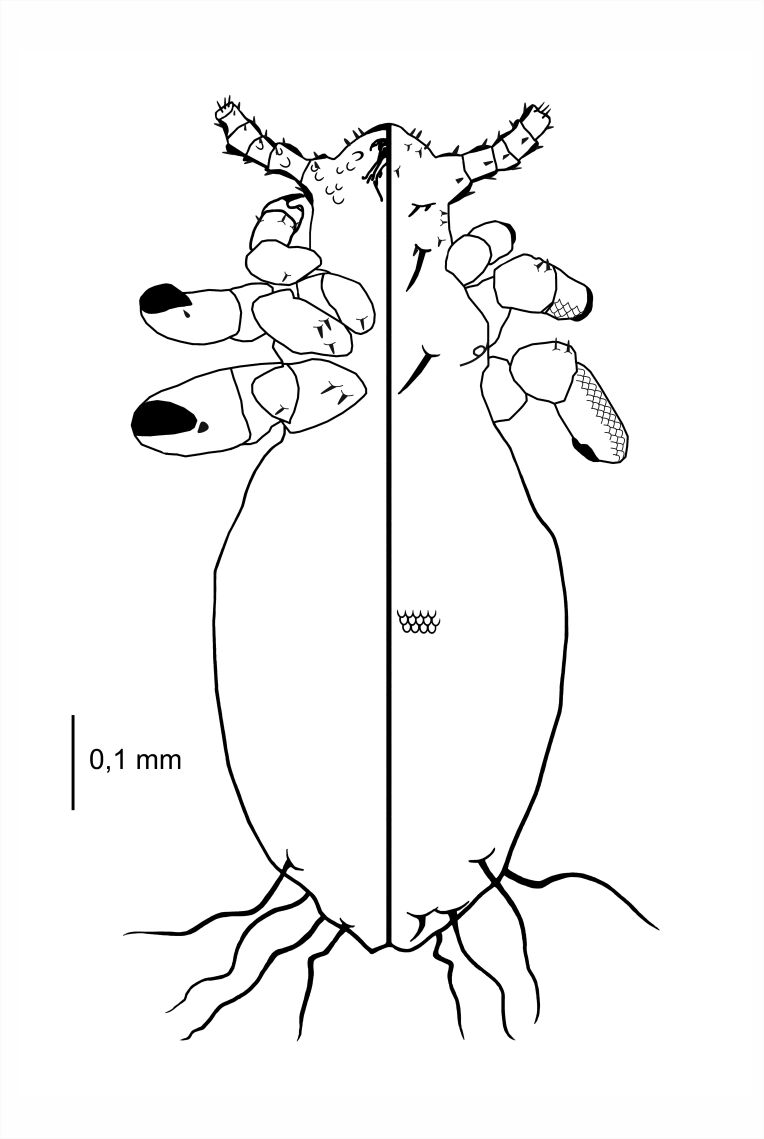
*Hoplopleura longula* nymph III, ventral (left) and dorsal (right) view.

**Figure 7.**
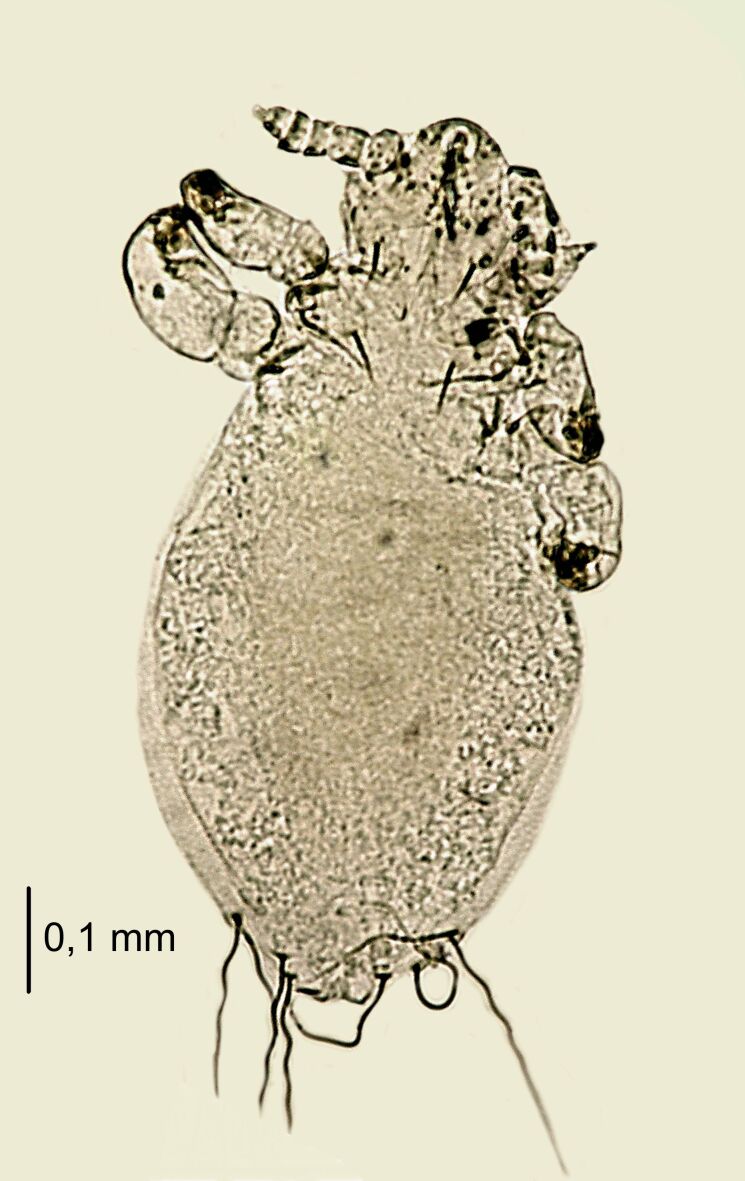
*Hoplopleura longula* nymph III, dorsal view.

**Nymph III** (Figs 6, 7) . Body of an adult, in the final stages of moulting, individual visible through the cuticle; body size sometimes smaller than in nymph II; body length: 0.87 mm (Table 2). **Head**. Wider than long. Ventral side: convex scales present, yet not as large as in nymph II; poorly visible on the head margins; VMHS, OS and AHS present; VPHS constitutes 50% of head length. Dorsal side: ISHS and OSHS present, similar length as in nymph II; PDHS long, reaching second segment of the abdomen, ADHS present. **Thorax**. Clearly visible borders between the thorax, head and abdomen. Dorsal side: DPTS reaching third segment of the thorax. **Abdomen**. Ovoid, elongated; MAS eight in number. After x-ray, sometimes adult individual visible (particular the plates and posterior part of the abdomen).

#### Biology

**Figure 8.**
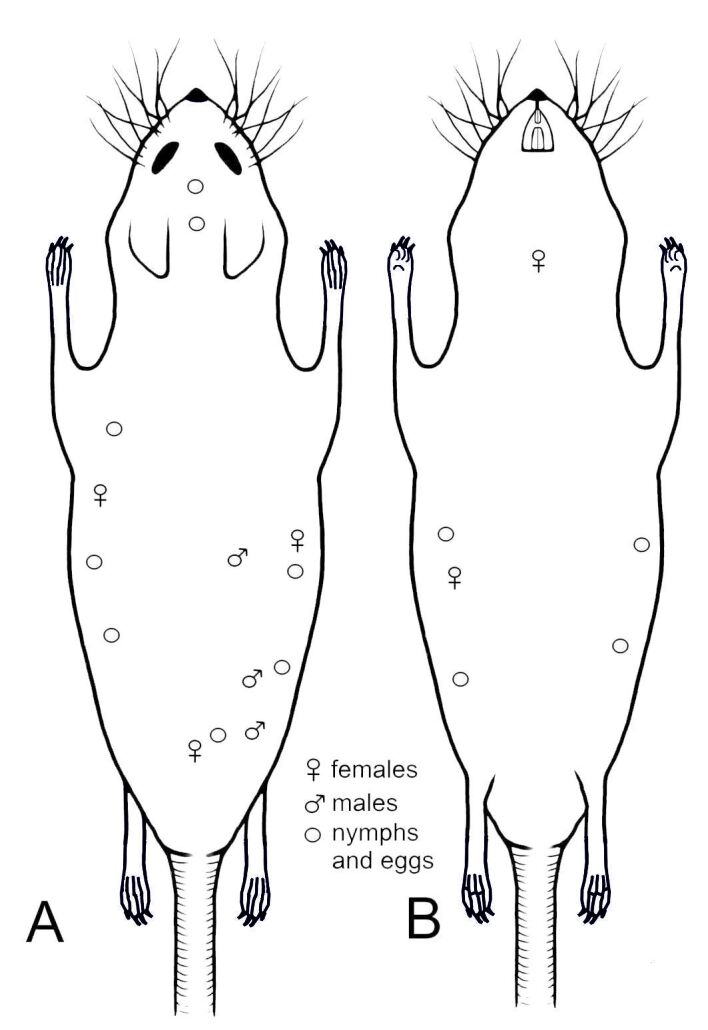
Topographic preferences of all stages of *Hoplopleura longula* on host’s body: A - dorsal and B - ventral view.

The lice demonstrated topographic preferences - a tendency for congregating along the sides of the host’s body and on both sides of the head (between the ears and on the neck) (Fig. 8)(Table 1).

## Discussion

In the present study, only *H. longula* was found amongst the lice. Despite the fact that there are also other species parasitising *M. minutus*, for example, of the genus *Polyplax* given in literature (Durden and Musser 1994), they were not found on the studied rodents.

The harvest mouse *M. minutus* is considered to be the main host of *H. longula*; it has not been recorded in any other hosts to date (Wegner 1966a, Krištofik and Lysy 1992).

Individual nymphs of *H. longula* are easily identifiable. Nymph I possessed four major abdominal setae and an elongated, ovoid abdomen. Nymph II possessed eight MAS and a barrel-shaped abdomen. Like nymph II, nymph III possessed eight MAS; however, its abdomen is elongated and ovoid. In addition, like nymph I, the body of the adult individual can be seen inside the nymph.

Regarding the location of the lice on the host, no comparative data are available in previous studies regarding the *H. longula*-*M. minutus* relationship. Despite this, similarities can be found with the distribution of *H. acanthopus* on *Microtus arvalis* (Dubinin 1953): in both cases, the lice were found on the head, the posterior part of the abdomen and on both sides of the host body; however, *H. longula* appeared to have a wider distribution on the sides and the dorsum on its rodent hosts. Similarly to *H. affinis* in *Apodemus agrarius* (Dubinin 1953), *H. longula* was also found on the dorsal portion of the head and on the nape of the animal. In both of the previous studies, Dubinin did not observe lice on the ventral portion of the head; this was also confirmed in the present study.

Current research indicates that *H. longula* is a rare species associated with only one, also rare host, *M. minutus.* This is the first description of all three nymphal stages of *H. longula*. This characteristic will support other researchers working on lice to make positive identifications which will benefit future research examining host-parasite associations.

## Supplementary Material

XML Treatment for Hoplopleura longula

## Figures and Tables

**Table 1.** Host specimens examined, louse life stages found and their location, with data on depositing in the Museum collections.

Host number	Host data (sex, collecting date, museum number)	Collected parasites	Parasites localisation on the host body	Museum specimens numbers
1	male, 15.07.1950, Bialowieza National Park	eggs	dorsal: left side of the body	-
2	male, 21.06.1949, Bialowieza National Park	eggs	dorsal: left side of the body	-
3	male, 01.08.1949, Bialowieza National Park	eggs	dorsal: tail area	-
4	male, 26.08.1949, Bialowieza National Park	eggs	dorsal: right side of the body	-
5	male, 08.09.1949, Bialowieza National Park	eggs	ventral: right side of the body	-
6	male, 06.11.1949, Bialowieza National Park	eggs	ventral: left side of the body	-
7	female, 07.01.1949, Bialowieza National Park	2??, 1?	? dorsal: right side of the body; ventral: right side of the body ? dorsal: right side of the body (closer to the centre)	UGDIZPMMmHHl1f, UGDIZPMMmHHl2f, UGDIZPMMmHHl1m,
8	male, 20.09.1949, Bialowieza National Park	2??, 1?, 1 N1	? ventral: neck area, right side of the body ? dorsal: right side of the body N1 dorsal: area between the ears	UGDIZPMMmHHl3f, UGDIZPMMmHHl4f, UGDIZPMMmHHl2m, UGDIZPMMmHHl1N1
9	male, 08.12.1949, Bialowieza National Park	1?, 1 N1, 1 N2	? dorsal: left side of the body and tail area N1, N2 ventral: left side of the body	UGDIZPMMmHHl5f, UGDIZPMMmHHl2N1, UGDIZPMMmHHl1N2
10	female, 07.10.1949, Bialowieza National Park	1?, 2 N3	? dorsal: right side of the body N3 dorsal: area between the eyes, left side of the body	UGDIZPMMmHHl3m, UGDIZPMMmHHl1N3 UGDIZPMMmHHl2N3
11	male, 25.08.1949, Bialowieza National Park	eggs	eggs dorsal: right side of the body; ventral: right side of the body	-

**Table 2.** Means and ranges of different immature stages of *Hoplopleura longula* [in mm].

Features	Nymph I [N = 2]	Nymph II [N = 1]	Nymph III [N = 2]
Head length	0.12 [0.12-0.12]	0.18	0.14 [0.13-0.14]
Head width	0.12 [0.11-0.12]	0.12	0.12 [0.11-0.12]
Thorax length	0.09 [0.09-0.10]	0.10	0.18 [0.12-0.23]
Thorax width	0.18 [0.17-0.20]	0.18	0.20 [0.19-0.22]
Abdomen length	0.32 [0.32-0.32]	0.51	0.56 [0.52-0.61]
Abdomen width	0.24 [0.23-0.24]	0.33	0.45 [0.41-0.48]
Whole body length	0.53 [0.53-0.54]	0.78	0.87 [0.87-0.88]

## References

[B6664433] Baker S. C, Whiting M., Johnson K. P., Murrel A. (2002). Phylogeny of the lice (Insecta, Phthiraptera) inferred from small subunit rRNA. Zoologica Scripta.

[B6664461] Buxton P. A. (1941). On the occurence of the crab-louse (*Phthirus
pubis*; Anoplura) in the hair of the head. Parasitology.

[B6664470] Cook E. F., Beer J. R. (1959). The immature stages of the genus *Hoplopleura* (Anoplura: Hoplopleuridae) in North America, with description of two new species. Journal of Parasitology.

[B6664480] Downs A. M.R., Stafford K. A., Coles G. C. (1999). Head lice: Prevalence in Schoolchildren and insecticide resistance. Parasitology Today.

[B6664489] Dubinin V. (1953). Parasite fauna of rodents and its changes in the delta of Volga. Parazitologicheskij Sbornik.

[B6664498] Durden L. A., Musser G. G. (1994). The mammalian hosts of the sucking lice (Anoplura) of the world: a host-parasite list. Bulletin of the Society for Vector Ecology.

[B6664507] Fagir D. M., Ueckermann E. A., Horak I. G., Bennett N. A., Lutermann H. (2014). The Namaqua rock mouse (*Micaelamys
namaquensis*) as a potential reservoir and host of arthropods vectors of diseases of medical and veterinary importance in South Africa. Parasites & Vectors.

[B6664517] Fisher R. I., Morton R. S. (1970). *Phthirus
pubis* infestation. The British Journal of Veneral Diseases.

[B6664543] Izdebska J. N., Fryderyk S., Buczek A., Blaszak Cz. (2010). New data of sucking lice (Phthiraptera: Anoplura) of rodents (Rodentia: Muridae, Cricetidae) in the northern Poland. Arthropods. Ecological and patological aspects of parasite – host relationship.

[B6664535] Izdebska J. N. (2014). Wszy. Poznaj i pokonaj problem.

[B6664562] Izdebska J. N., Kozina P., Cierocka K., Mierzynski L., Buczek A., Blaszak Cz. (2018). Human lice *Pediculus
humanus* and pediculosis in the past and present - occurrence, diagnostics and controlling. Arthropods. At the beginning of the new Century.

[B6664575] Johnson P. T. (1960). The Anoplura of African rodents and insectivores. Technical Bulletin.

[B6664584] Johnson P. T. (1972). Some Anoplura from Oriental region. A study of *Hoplopleura
pacifica* Ewing and Allies. Journal of Medical Entomology.

[B6664593] Kadulski S., Izdebska J. N., Buczek A., Blaszak Cz. (2006). Methods used in studies of parasitic arthropods in mammals. Arthropods. Epidemiological importance.

[B6664606] Kim K. C. (1966). A new species of *Hoplopleura* from Thailand, with notes and description of nymphal stages of *Hoplopleura
captiosa* Johnson (Anoplura). Parasitology.

[B6664628] Kim K. C., Ludwig H. W. (1978). The family classification of the Anoplura. Systematic Entomology.

[B6664615] Kim K. C., Morand S., Krasnov B. R., Poulin R. (2006). Blood sucking lice (Anoplura) of small mammals: True parasites. Micromammals and microparasites from evolutionary to management.

[B6664655] Krcmar S., Trilar T. (2017). The blood sucking lice (Phthiraptera: Anoplura) of Croatia: review and new data. Turkish Journal of Zoology.

[B6664646] Krištofik J., Lysy J. (1992). Seasonal dynamics of sucking lice (Anoplura) in small mammals (Insectivora, Rodentia) in the natural foci of infections in South West Slovakia. Biologia.

[B6664637] Krištofik J. (1999). Sucking lice (Phthiraptera) from Mongolian mammals. Biologia.

[B6664664] Levot G. (2000). Resistance and control of lice on humans and production animals. International Journal of Parasitology.

[B6664673] Mey E. (2003). On the development of animal louse systematics (Insecta, Phthiraptera) up to the present day. Rudolfstädter Naturhistorische Schriften.

[B6664682] Müller F. (1949). Das Zahlenverhältnis der Geschlechter in Zuchtpopulationen der Kleiderlaus (*Pediculus
corporis* de Geer, Anoplura). Zeitschrift für Parasitenkunde.

[B6664691] Piotrowski F. (1961). Morphogenesis of the pathways of the sexual system of human lice *Pediculus
humanus* L. (Anoplura).. Acta Zoologica Cracoviensis.

[B6664709] Piotrowski F., Wegner Z., Zóltkowski Z. (1976). Division: Insects (Insecta) cd. Order: Sucking lice (Anoplura). Arachnoentomologia lekarska.

[B6664700] Piotrowski F. (1998). News about biology and combating Anoplura. Wiadomosci Parazytologiczne.

[B6664723] Robinson D., Leo N., Prociv P., Baker S. C. (2003). Potencial role of head lice, *Pediculus
humanus
capitis*, as vector of *Rickettsia
prowazekii*. Parasitology Research.

[B6664732] Roux V., Raoult D. (1999). Body Lice as Tools of Diagnosis and Surveillance of Reemerging Diseases. Journal of Clinical Microbiology.

[B6664741] Weaver H. J., Barton P. S. (2008). A new species of sucking louse (Phthiraptera: Anoplura) from Australia, and a key to the Australian species of *Hoplopleura*. Zootaxa.

[B6664750] Wegner Z. (1966). The immature stages of the louse *Hoplopleura
captiosa* Johnson syn. *Hoplopleura
musculi* Wegner.. Bulletin of the Institute of Maritime and Tropical Medicine.

[B6664776] Wegner Z., Jaczewski T. (1966). Wszy. Anoplura. Katalog Fauny Polski.

[B6664759] Zhang Z. Q. (2018). Repositories for mite and tick specimens: acronyms and their nomenclature. Systematic and Applied Acarology.

